# Supplementary material for: Individual light history matters to deal with the Antarctic summer
Source: Sci Rep. 2023 Jul 26;13:12081. doi: 10.1038/s41598-023-39315-y (PMC10372057; doi:10.1038/s41598-023-39315-y)
Supplement: Supplementary file 1 — Supplementary Information. [file 41598_2023_39315_MOESM1_ESM.pdf]

621

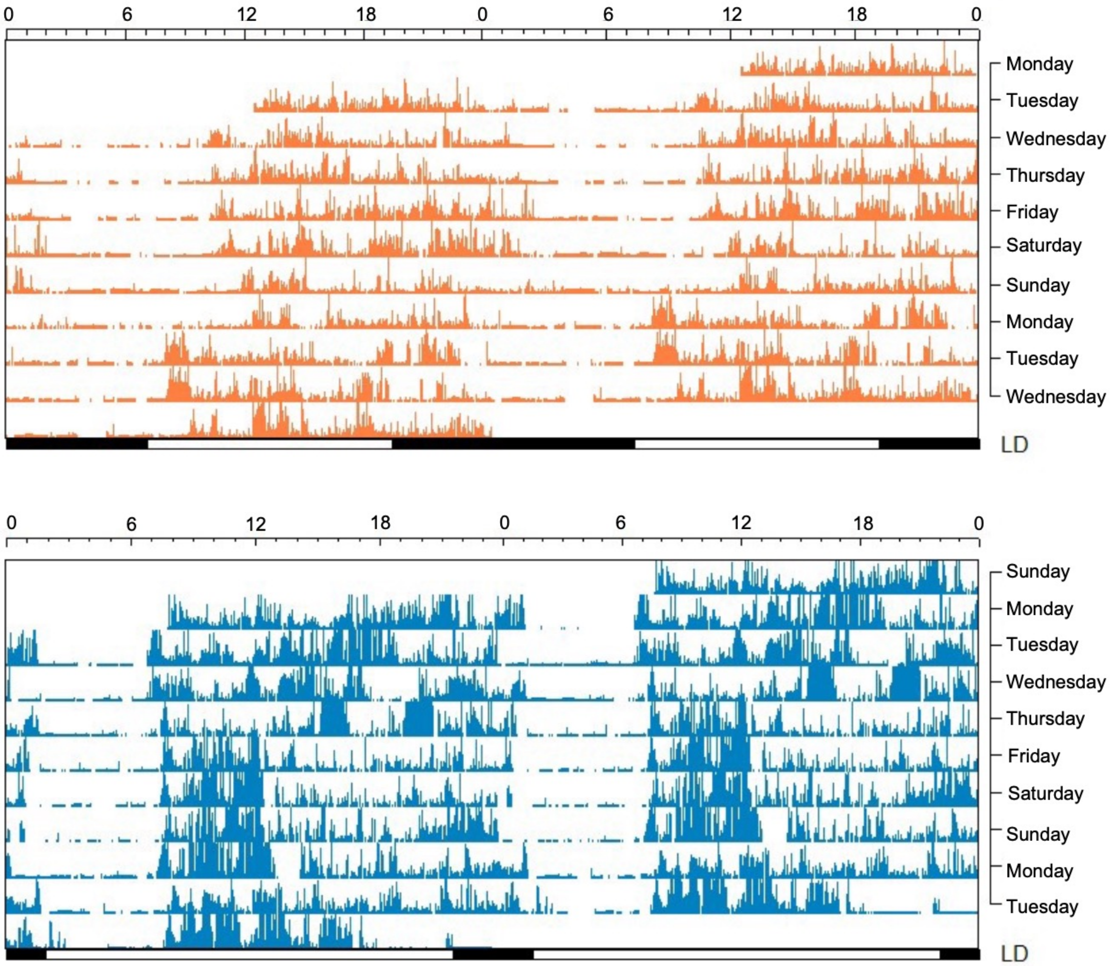
0 6 12 18 0 6 12 18 0
Monday
Tuesday
Wednesday
Thursday
Friday
Saturday
Sunday
Monday
Tuesday
Wednesday
LD
0 6 12 18 0 6 12 18 0
Sunday
Monday
Tuesday
Wednesday
Thursday
Friday
Saturday
Sunday
Monday
Tuesday
LD

622

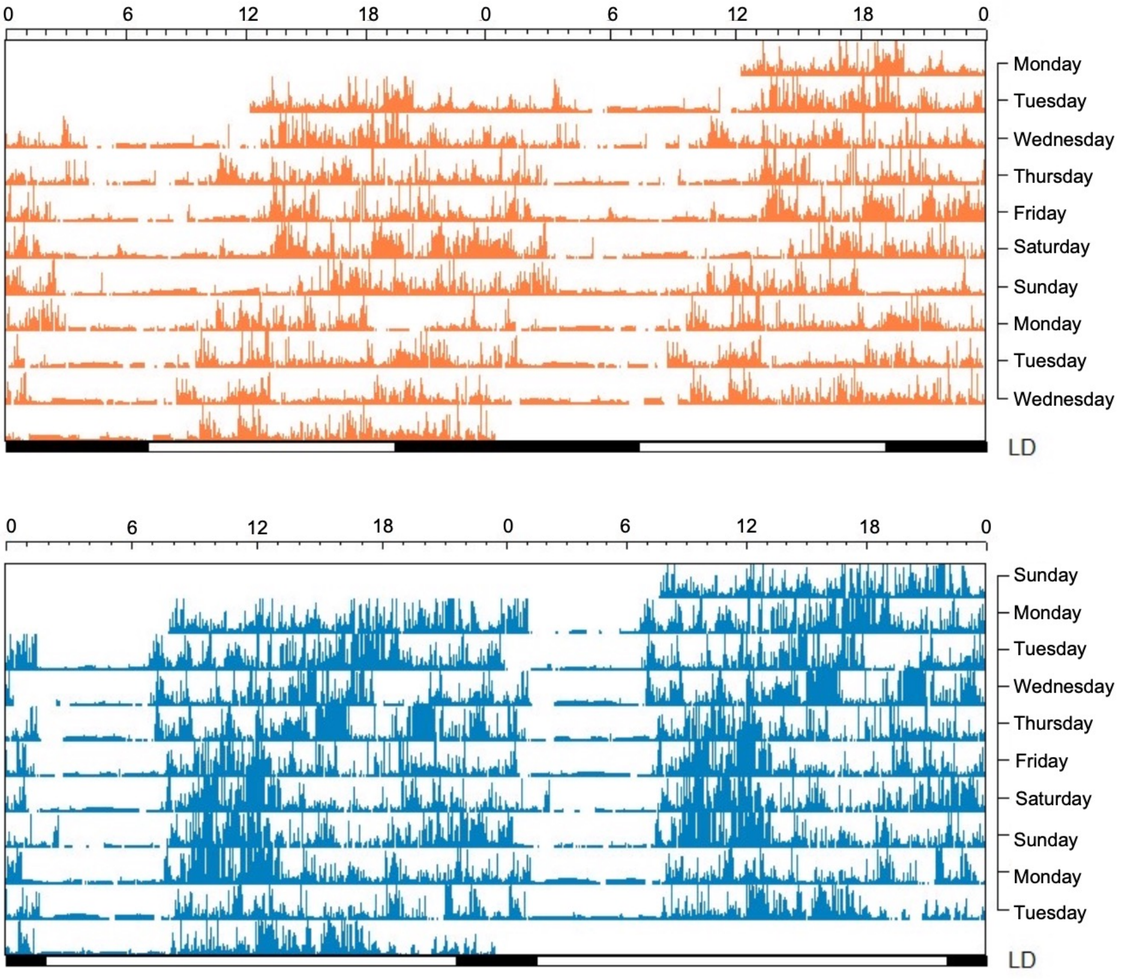
0 6 12 18 0 6 12 18 0
Monday
Tuesday
Wednesday
Thursday
Friday
Saturday
Sunday
Monday
Tuesday
Wednesday
LD
0 6 12 18 0 6 12 18 0
Sunday
Monday
Tuesday
Wednesday
Thursday
Friday
Saturday
Sunday
Monday
Tuesday
LD

623

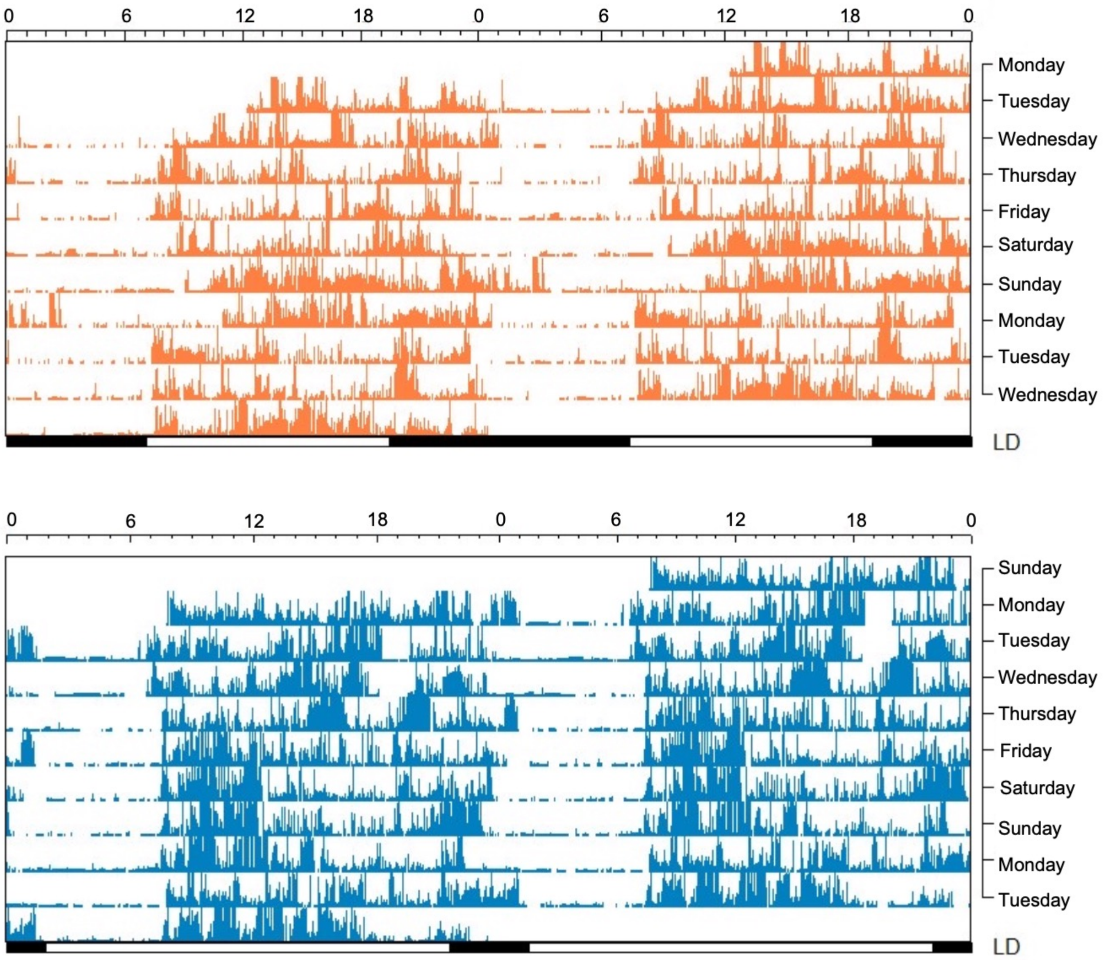
0 6 12 18 0 6 12 18 0
Monday
Tuesday
Wednesday
Thursday
Friday
Saturday
Sunday
Monday
Tuesday
Wednesday
LD
0 6 12 18 0 6 12 18 0
Sunday
Monday
Tuesday
Wednesday
Thursday
Friday
Saturday
Sunday
Monday
Tuesday
LD

624

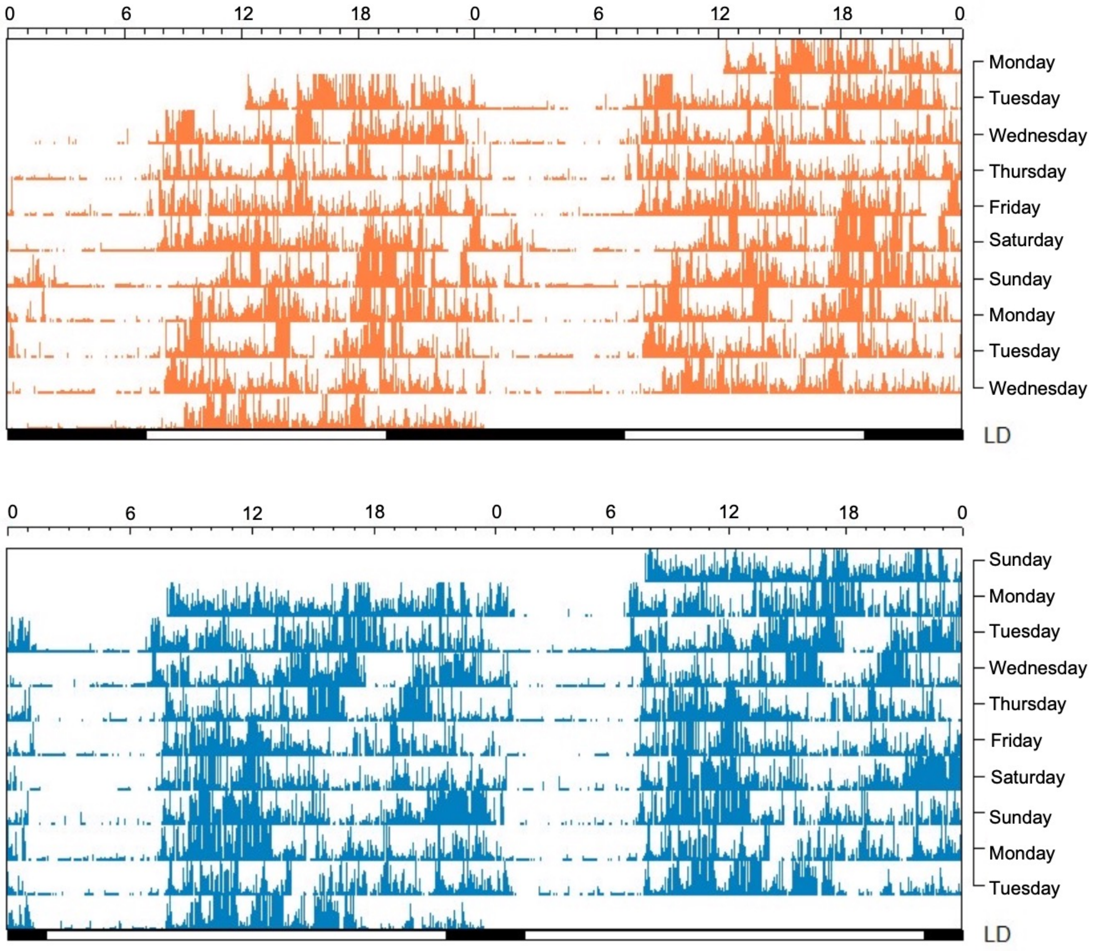
0 6 12 18 0 6 12 18 0
Monday
Tuesday
Wednesday
Thursday
Friday
Saturday
Sunday
Monday
Tuesday
Wednesday
LD
0 6 12 18 0 6 12 18 0
Sunday
Monday
Tuesday
Wednesday
Thursday
Friday
Saturday
Sunday
Monday
Tuesday
LD

625

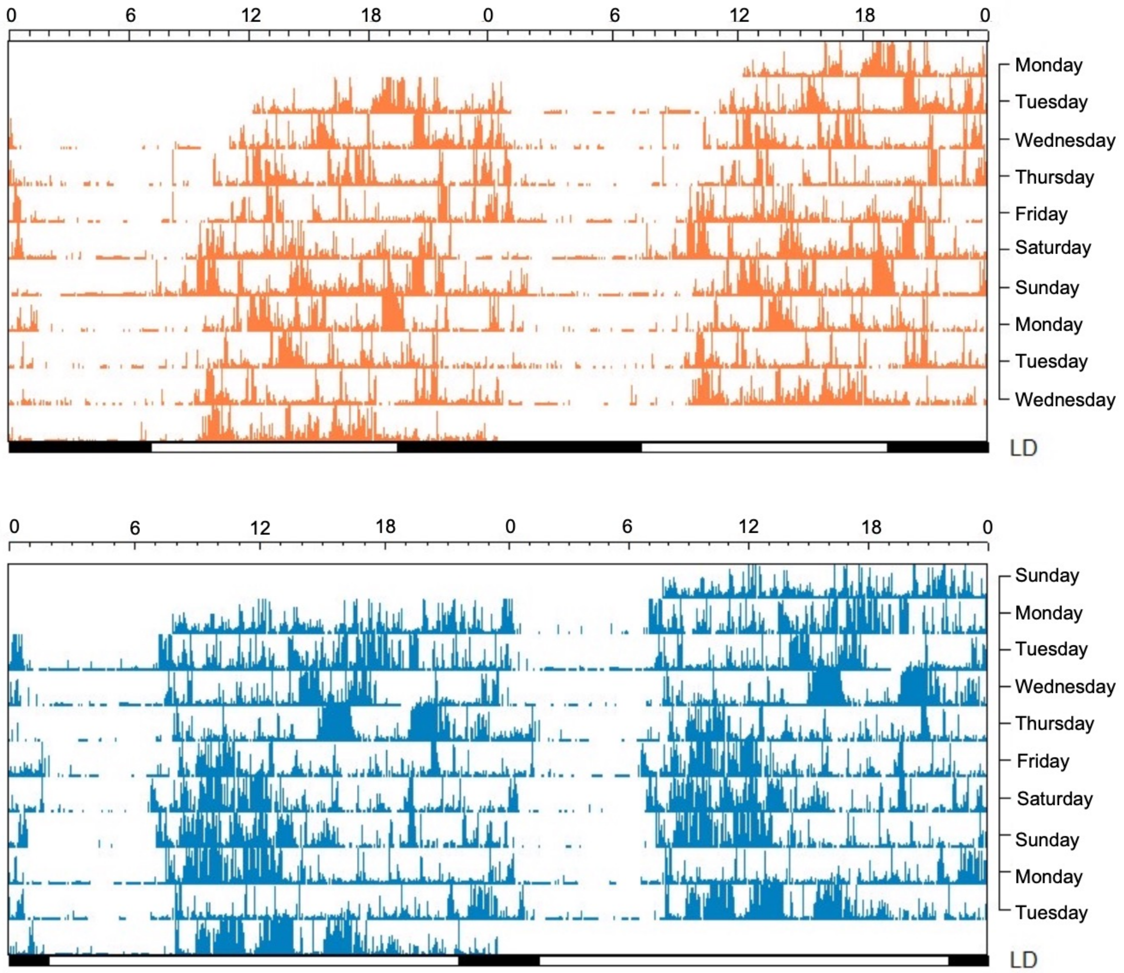
0 6 12 18 0 6 12 18 0
Monday
Tuesday
Wednesday
Thursday
Friday
Saturday
Sunday
Monday
Tuesday
Wednesday
LD
0 6 12 18 0 6 12 18 0
Sunday
Monday
Tuesday
Wednesday
Thursday
Friday
Saturday
Sunday
Monday
Tuesday
LD

626

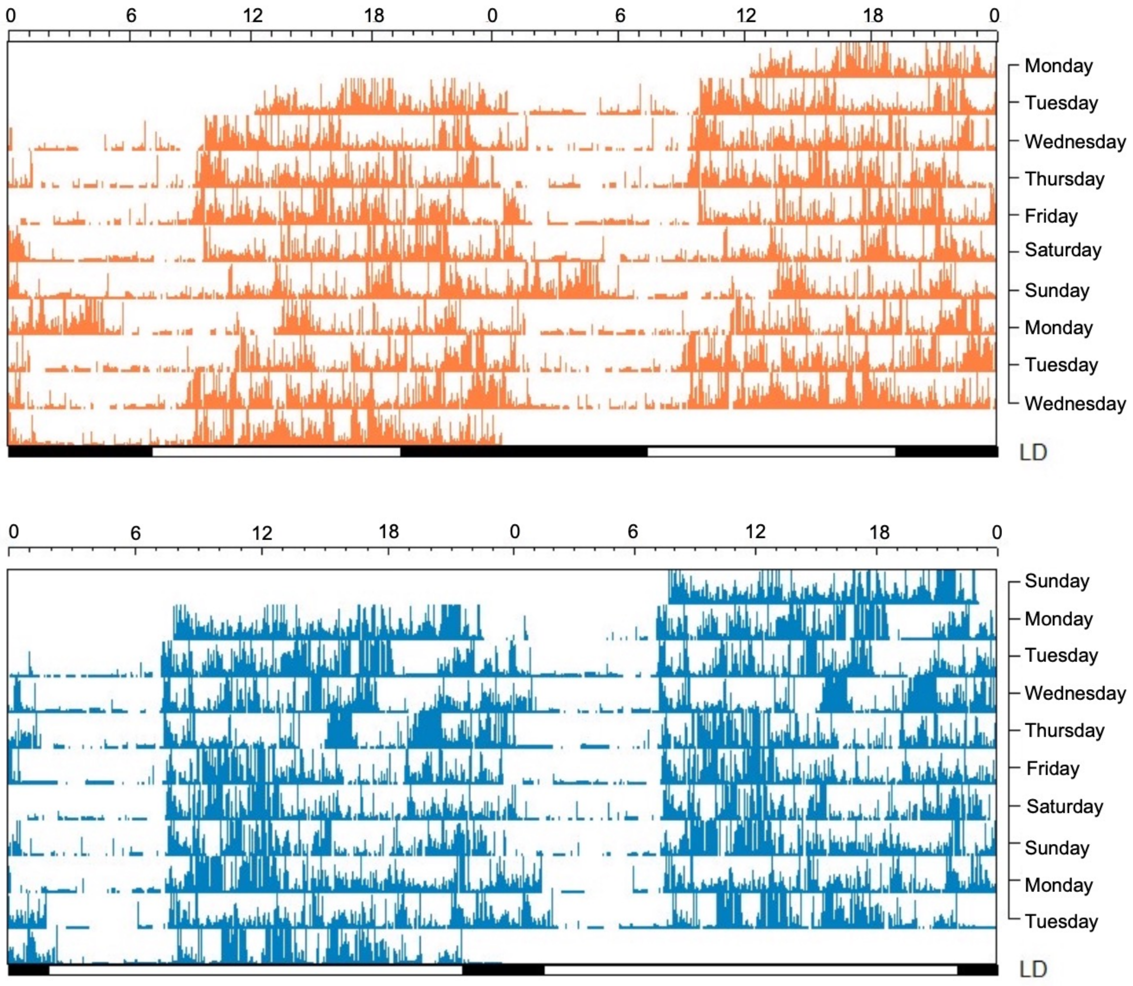
0 6 12 18 0 6 12 18 0
Monday
Tuesday
Wednesday
Thursday
Friday
Saturday
Sunday
Monday
Tuesday
Wednesday
LD
0 6 12 18 0 6 12 18 0
Sunday
Monday
Tuesday
Wednesday
Thursday
Friday
Saturday
Sunday
Monday
Tuesday
LD

627

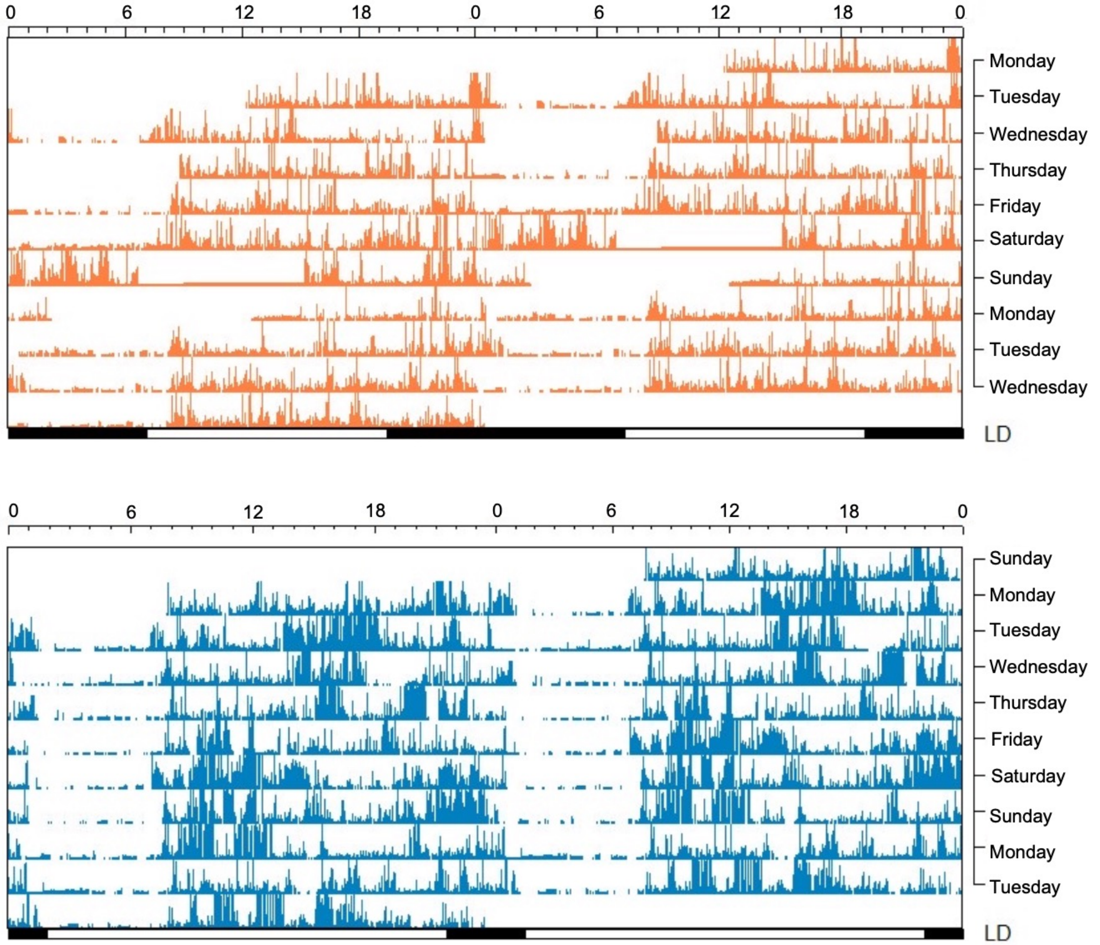
0 6 12 18 0 6 12 18 0
Monday
Tuesday
Wednesday
Thursday
Friday
Saturday
Sunday
Monday
Tuesday
Wednesday
LD
0 6 12 18 0 6 12 18 0
Sunday
Monday
Tuesday
Wednesday
Thursday
Friday
Saturday
Sunday
Monday
Tuesday
LD

638

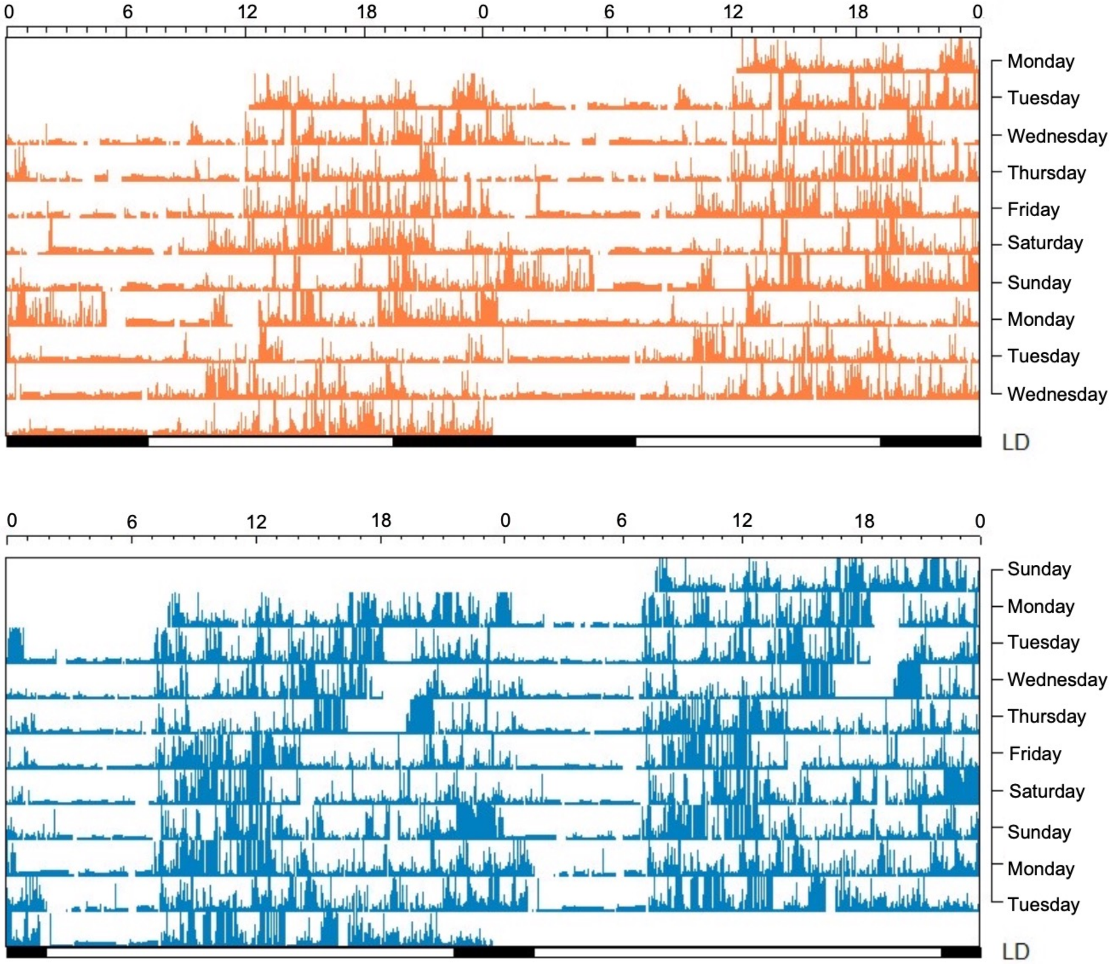
0 6 12 18 0 6 12 18 0
Monday
Tuesday
Wednesday
Thursday
Friday
Saturday
Sunday
Monday
Tuesday
Wednesday
LD
0 6 12 18 0 6 12 18 0
Sunday
Monday
Tuesday
Wednesday
Thursday
Friday
Saturday
Sunday
Monday
Tuesday
LD

639

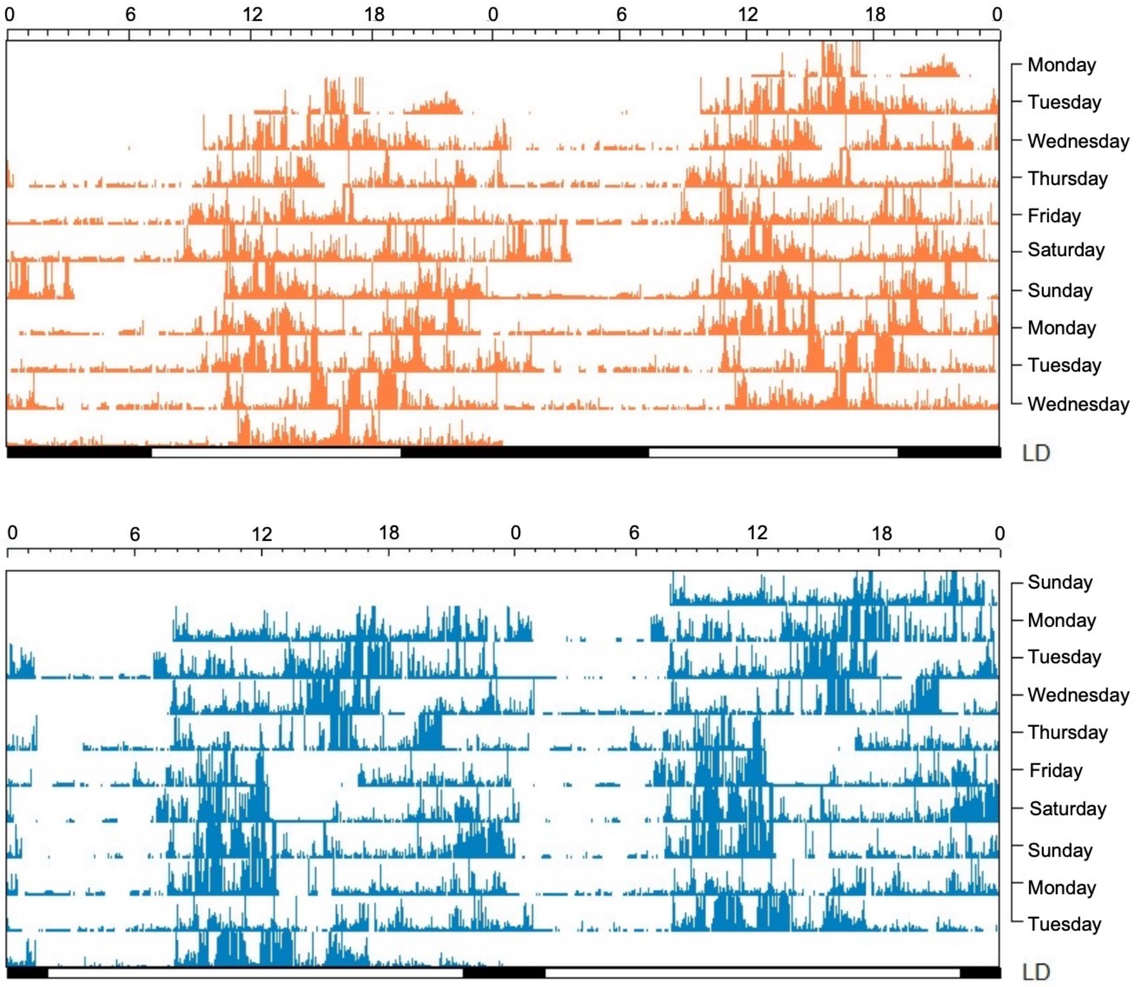
0 6 12 18 0 6 12 18 0
Monday
Tuesday
Wednesday
Thursday
Friday
Saturday
Sunday
Monday
Tuesday
Wednesday
LD
0 6 12 18 0 6 12 18 0
Sunday
Monday
Tuesday
Wednesday
Thursday
Friday
Saturday
Sunday
Monday
Tuesday
LD

640

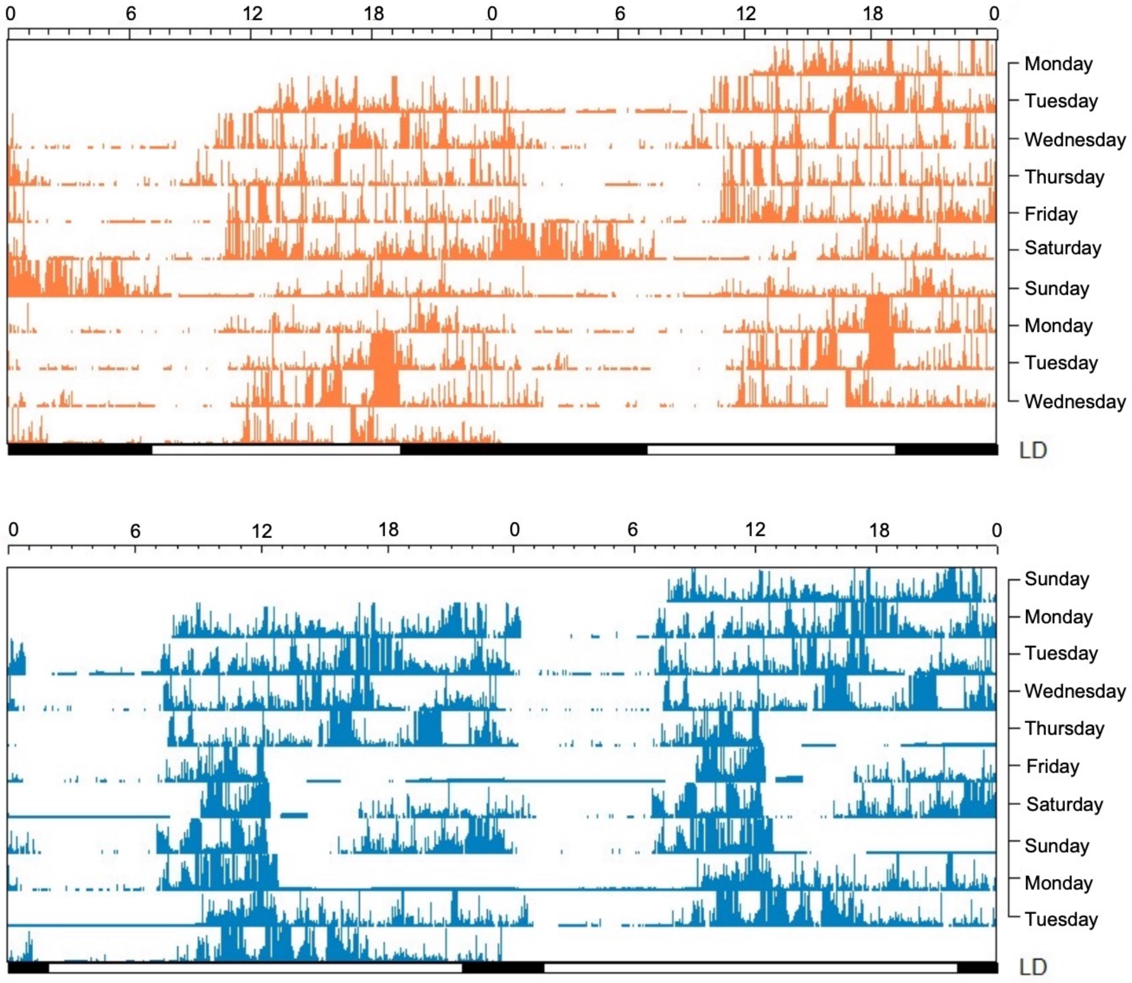
0 6 12 18 0 6 12 18 0
Monday
Tuesday
Wednesday
Thursday
Friday
Saturday
Sunday
Monday
Tuesday
Wednesday
LD
0 6 12 18 0 6 12 18 0
Sunday
Monday
Tuesday
Wednesday
Thursday
Friday
Saturday
Sunday
Monday
Tuesday
LD

641

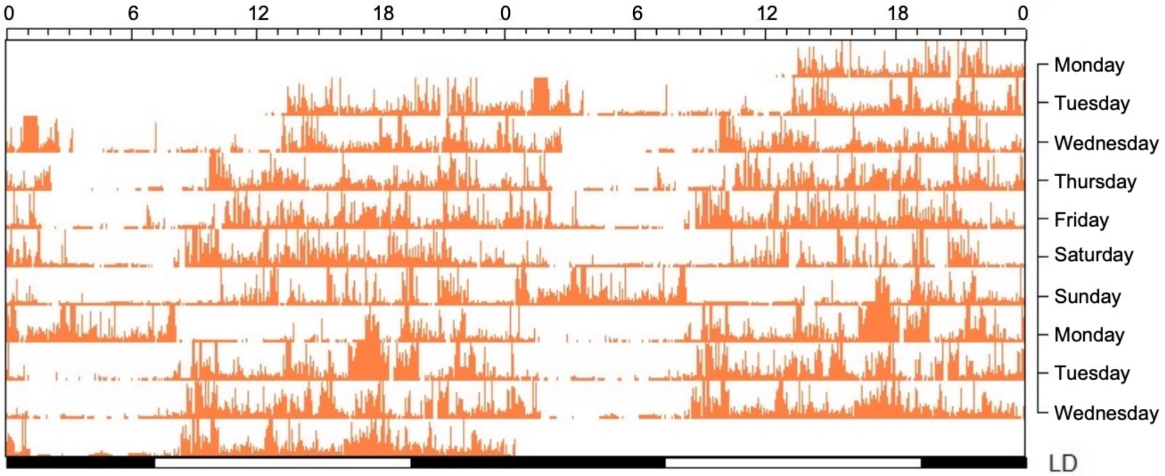

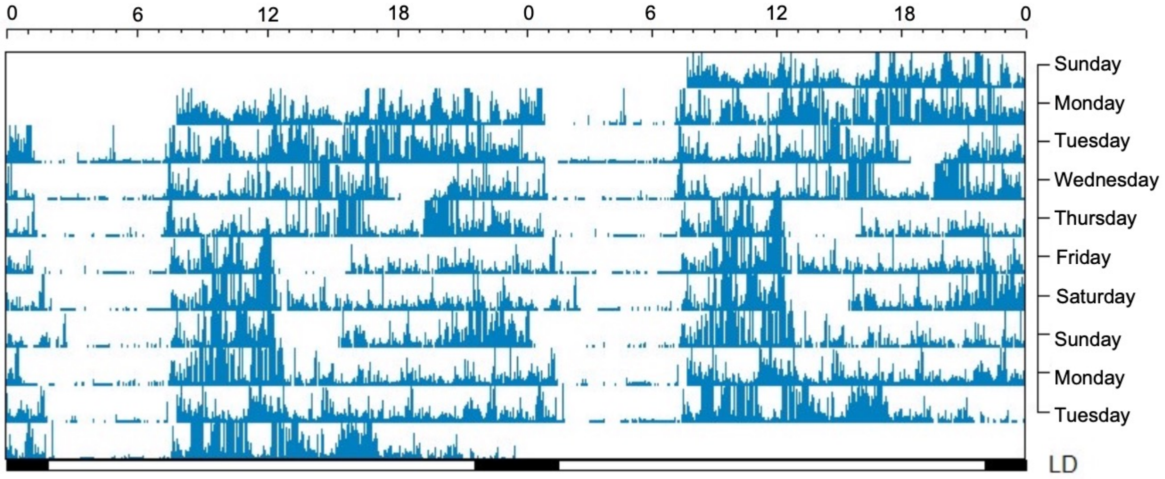

Supplementary material

Figure S1: Activity actograms for all the participants (n = 11) during the 10 consecutive days of recording for both locations: Montevideo (MVD - top, orange) and Antarctica (ANT - below, blue). Data are presented in rows of 48 h, bars at the bottom indicate light-dark cycle, scale on top indicates time in hours.

Table S1 - Absolute light exposure per participant in the two light-sensitive circadian phase windows: the phase-advance window (PAW) and the phase-delay window (PDW) in both location (ANT and MVD) and the difference between the average light exposure in both the PDW (Δ light exposure PDW= light exposure PDW_{ANT} - light exposure PDW_{MVD}) and the PAW (Δ light exposure PAW= light exposure PAW_{ANT} - light exposure PAW_{MVD}) for each participant.

	**Light exposure phase-advance window (PAW, lux)**			**Light exposure phase-delay window (PDW, lux)**		
**#participant**	**ANT**	**MVD**	**Δ light exposure**	**ANT**	**MVD**	**Δ light exposure**
621	2615	340	2275	254	19	236
622	6026	1617	4409	106	94	12
623	275	561	-286	577	183	394
624	251	414	-163	801	293	508
625	926	4	922	318	50	268
626	1251	277	973	497	32	466
627	595	705	-110	201	163	38
638	4156	584	3572	346	46	300
639	2312	258	2054	398	43	355
640	5885	457	5428	22	11	11
641	405	214	190	839	184	655

Table S2: Linear model fitting was performed using the most recent shell of Rstudio (version: 2023.03.0386) and the “nlme” R-package for linear and non-linear Mixed-Effects Modeling (Pinheiro J, Bates D, DebRoy S, Sarkar D, R Core Team (2021) nlme: Linear and Nonlinear Mixed Effects Models_. R package version 3.1-152, URL: https://CRAN.R-project.org/package=nlme>). For all models, participant ID was a random effect and included as intercept. A critical p-value of 0.05 was maintained for all analyses. Mid-sleep was included in the model as a dependent variable, and light levels in PAW and PDW were included as fixed effects. When fitting the models, Akaike Information Criteria (AIC) were compared, selecting the one with the lowest AIC value that included all mixed effects parameters (shown in bold). 78 observations.

Model	AIC
MS.ANT ~ PAW.ANT * PDW.ANT	263.676
**MS.ANT ~ PAW.ANT + PDW.ANT**	**262.136**
MS.MVD ~ PAW.MVD * PDW.MVD	291.642
**MS.MVD ~ PAW.MVD + PDW.MVD**	**289.971**
